# The future of subalpine forests in the Southern Rocky Mountains: Trajectories for *Pinus aristata* genetic lineages

**DOI:** 10.1371/journal.pone.0193481

**Published:** 2018-03-19

**Authors:** Sparkle L. Malone, Anna W. Schoettle, Jonathan D. Coop

**Affiliations:** 1 Department of Biological Sciences, Florida International University, Miami, Florida, United States of America; 2 USDA Forest Service, Rocky Mountain Research Station, Fort Collins, Colorado, United States of America; 3 Biology, Western State Colorado University, Gunnison, Colorado, United States of America; Chinese Academy of Forestry, CHINA

## Abstract

Like many other high elevation alpine tree species, Rocky Mountain bristlecone pine (*Pinus aristata* Engelm.) may be particularly vulnerable to climate change. To evaluate its potential vulnerability to shifts in climate, we defined the suitable climate space for each of four genetic lineages of bristlecone pine and for other subalpine tree species in close proximity to bristlecone pine forests. Measuring changes in the suitable climate space for lineage groups is an important step beyond models that assume species are genetically homogenous. The suitable climate space for bristlecone pine in the year 2090 is projected to decline by 74% and the proportional distribution of suitable climate space for genetic lineages shifts toward those associated with warmer and wetter conditions. The 2090 climate space for bristlecone pine exhibits a bimodal distribution along an elevation gradient, presumably due to the persistence of the climate space in the Southern Rocky Mountains and exclusion at mid-elevations by conditions that favor the climate space of other species. These shifts have implications for changes in fire regimes, vulnerability to pest and pathogens, and altered carbon dynamics across the southern Rockies, which may reduce the likelihood of bristlecone pine trees achieving exceptional longevity in the future. The persistence and expansion of climate space for southern bristlecone pine genetic lineage groups in 2090 suggests that these sources may be the least vulnerable in the future. While these lineages may be more likely to persist and therefore present opportunities for proactive management (e.g., assisted migration) to maintain subalpine forest ecosystem services in a warmer world, our findings also imply heighted conservation concern for vulnerable northern lineages facing range contractions.

## Introduction

Tree species are increasingly threatened by anthropogenic environmental change including deforestation [1], native and introduced insects and pathogens [2], altered disturbance regimes [3,4], and climate change [5–7]. Climate is a crucial factor controlling where tree species occur [8] and as the climate changes so then do species distributions. Studies have shown that many tree species respond to shifts in climate by migrating [9–12], which influences ecosystem structure and function with the potential to produce novel ecosystems [13].

Predicting species range shifts in response to climate change is a major challenge in conservation. A species is composed of a compilation of genetically differentiated populations with clinal variation along environmental gradients [14]. These populations may respond differently to climate change [15] and the species-wide genetic structure may change [16] as coverage by some populations contract and others expand and evolve [16,17]. Modeling the future distribution of suitable climate space for genetically divergent populations within a species can identify those that may be disproportionately represented or underrepresented in the future and therefore of current management or conservation interest.

Some of the most vulnerable ecosystems to climate change include subalpine forests [11,18] where growing space declines with elevation and species distributions are defined by distinct climatic gradients (altitudinal) and microclimate effects (i.e. topography, aspect, soil). Climate change is projected to be rapid and heightened in these habitats, where subalpine treelines are expected to move up in elevation with warming growing season temperatures [11]. To understand the potential vulnerability to climate change for long lived high-elevation species, we can evaluate changes in the suitable climate space for tree species. Suitable climate space is determined based on empirical relationships between observed species distributions and climate [19].

In the southern Rocky Mountains, Rocky Mountain bristlecone pine (*Pinus aristata*; henceforth, bristlecone pine) is a long-lived tree species (>500 years) [20] of high elevation forests (2750 to 3670 m elevation) where it occupies sites beyond the physiological limits of most other tree species. Co- and sub-dominant species of bristlecone pine forests include Engelmann spruce (*Picea engelmannii* Parry ex Engelm.), Douglas-fir (*Pseudotsuga menziesii* (Mirbel) Franco), subalpine or corkbark fir (*Abies lasiocarpa* (Hook.) Nutt.), limber pine, quaking aspen (*Populus tremuloides* Michx.), ponderosa pine (*Pinus ponderosa* Lawson), and, rarely, pinyon pine (*Pinus edulis* Engelm.). Although bristlecone pine has persisted through historical shifts in climate, a combination of genetic (i.e. low genetic diversity and limited gene flow), demographic (i.e., long generation times and low regeneration rates), and ecological (i.e., forest composition, disease, and insect outbreaks) characteristics suggest that it may be particularly vulnerable to contemporary and projected climate change [21].

Consistent with its narrow distribution, bristlecone pine has the lowest genetic diversity of the high-elevation five-needle pines [22] and is structured into populations with varying proportions of three genetic lineages that differ in diversity and associated climatic factors [22]. Lineage 1 is the most common and present throughout the range and some populations also contain significant proportions of lineage 2 or 3 [22]. Hereafter, populations dominated or solely composed of lineage 1 are referred to as lineage group 1, while those with either lineage 2 or 3 are referred to as lineage group 2 or lineage group 3, respectively. Lineage group 3 has the highest genetic diversity of the three groups and is most common on sites towards the northeastern and southeastern edges of the bristlecone pine core distribution. These sites are the warmest and wettest compared with sites occupied by the other two groups [22].

Although long-lived tree species have adapted repeatedly to past climate change [23], when tolerance limits are exceeded, survival is dependent on the species’ phenotypic plasticity, ability to genetically adapt, or migrate to an area with a suitable climate space. Although alpine treelines have moved in the past in response to changing conditions [24,25], the rate of climate change in projections for the near future in many alpine areas is much greater than the rate of change in historical periods [26]. Climate induced range shifts are generally poleward or upward, and the rate of loss from the trailing edge of a species range is likely to exceed the rate of expansion at the leading edge [12]. As growing space declines with elevation, high elevation ecosystems are likely to see changes in species composition as species migrate up slope.

The objective of this research is to use bristlecone pine population genetics, geographic distribution, and suitable climate space to quantify the species vulnerability to climate change in the near future (the year 2090). Specifically, we (1) evaluate changes in the suitable climate space for bristlecone pine, (2) determine the potential role of population genetic structure (i.e. genetic lineage group) in defining the suitable climate space, and (3) quantify the potential for range shifts in neighboring species and forest types. We hypothesize that, in the near future, (1) the climate space for bristlecone pine will decline; (2) due to its greater climate variability and associated genetic variation, the climate space for lineage group 3 will be more extensive than the climate space for groups 1 or 2; and (3) the climate space for lower elevation species will encroach on the area currently occupied by bristlecone pine.

## Materials and methods

### Study area

The core of the bristlecone pine range is in south-central Colorado and extends south into New Mexico along the Sangre de Cristo Mountains, with a disjunct population on the San Francisco Peaks in central Arizona [22, 27]. Common bristlecone pine forest types include: (1) sub-dominant spruce and fir species, (2) sub-dominant spruce and fir with aspen, (3) co-dominant limber pine, (4) dominant non-regenerating bristlecone pine, and (5) dominant spruce and fir species with a sub-dominant bristlecone pine. The northern edge of the bristlecone pine range coincides with the northern reach of the North American Monsoon, suggesting a dependence on mid- to late-summer precipitation [28]. This pioneer species primarily regenerates following fire and variation in forest structure is often driven by time since fire [29], although within-forest gaps can also support regeneration [30]. As time since fire increases, bristlecone pine can be replaced by other species over long-time periods on mesic sites.

### Tree species location and genetic lineage data

Data from multiple sources were utilized. Forest Inventory and Analysis (FIA) plots were used to determine where all species of interest were present or absent. The USDA Forest Service maintains these permanent plots that systematically sample woody vegetation on forested and non-forested lands within the United States [32,33]. There are 162,409 FIA plots in the geographic area of interest (Arizona, Colorado, New Mexico, Utah, and Wyoming) with bristlecone pine occurring at 235 of these plots. The FIA datasets include a tabulation of the presence-absence of species and elevation. In accordance with FIA proprietary restrictions, true-plot locations are not available for publication. We used the publicly available FIA data supplemented with an additional 165 plot locations throughout the range of bristlecone pine [21, 34]. To understand how the true-plot vs publicly available plot locations might influence our results, we compared (1) the measured true FIA plot elevations to the publicly released plot elevations (i.e. public-plot location) and (2) the climate space defined by the true- and public-plot locations (S1 Fig). Differences in elevation ranged from 0.3 meters to 276 meters and 80% of the public-plot elevations were within 100 meters of the measured elevation of the true-plots. The climate space defined by Crookston et al. [35], who used the true FIA plots locations, was substantially smaller (72%) and exhibited 86% overlap with the climate space defined with the public FIA location data and supplemental plots. The climate space for the public FIA and supplemental plots captured the range and distribution of the Rocky Mountain Bristlecone pine trees. Studies of similar comparisons have also shown that the underlying distributions of predictor variables sampled with true versus public coordinates are not significantly different at any extent [36].

**Fig 1:**
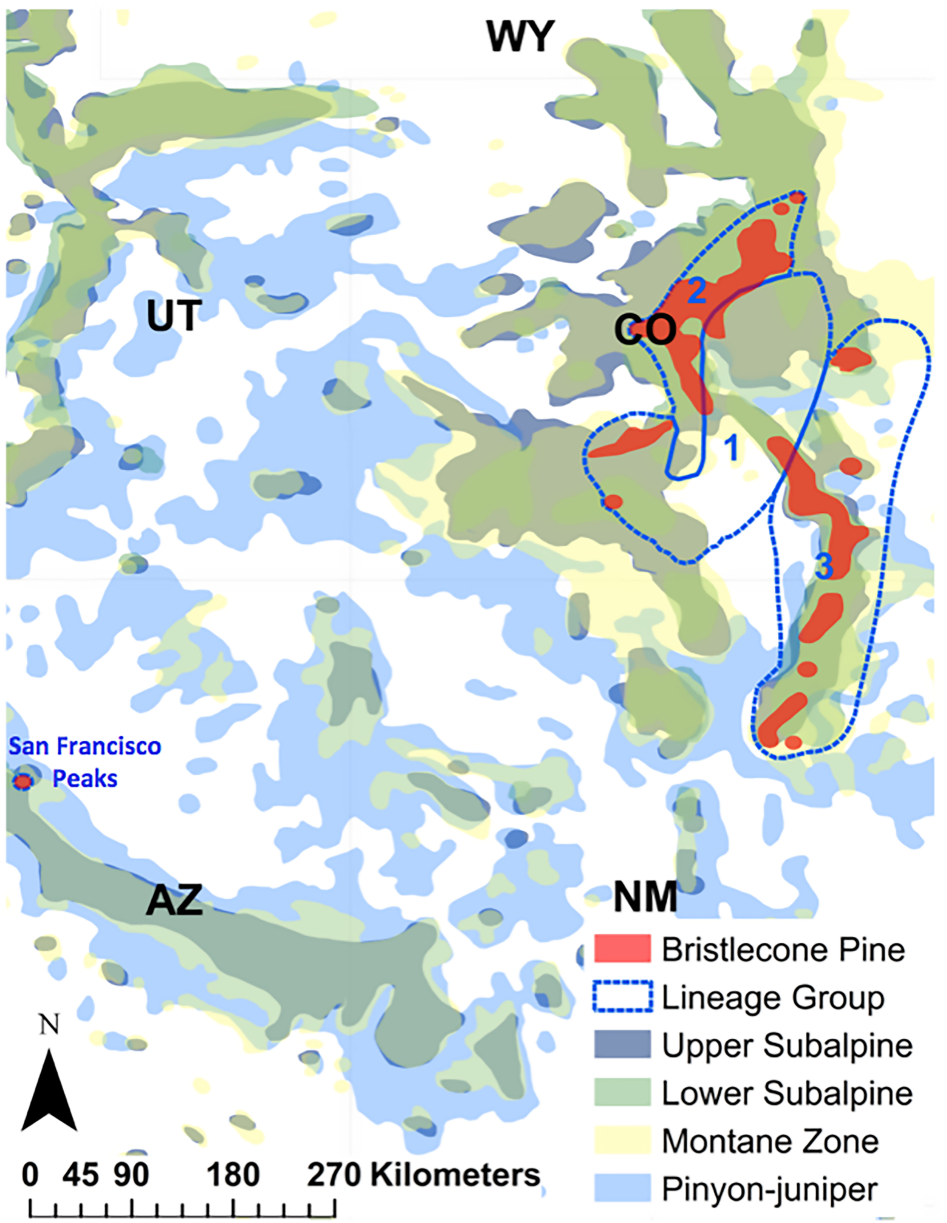
Subalpine and montane species range maps based on Little [31]. Subalpine species include lower subalpine (limber pine, lodgepole pine) and upper subalpine species (Engelmann spruce, subalpine fir, and Douglas fir). Ponderosa pine and quaking aspen make up the montane zone while pinyon-juniper represents lower elevation woodland species. The numbers (1, 2, and 3) in the delineated areas refer to the genetic lineage groups for Rocky Mountain bristlecone pine (red) [22].

The geographic location of the bristlecone pine lineage groups defined in Schoettle et al. [22] was used to assign a lineage group to all plots that contained bristlecone pine. Schoettle et al. [22] identified these dominant bristlecone pine genetic lineages. Genetic patterns were evaluated at sixteen sites across the four main mountain ranges (i.e., the Sawatch-Mosquito Range, the Front Range, the San Juan Mountains, and the Sangre de Cristo Mountains) that are in the core bristlecone pine distribution. Sites represented the elevational, latitudinal, and climatic gradients of the species. Genetic diversity was assessed with 21 isozyme loci and sampled trees were assigned to three genetic lineages that varied in diversity and admixture [22]. Geographic lineage group was determined by the proportion of trees in the three lineages. In lineage group 1, the majority of the trees were assigned to lineage 1. Lineage groups 2 and 3 included those sites with a higher proportion of individuals assigned to lineages 2 and 3, respectively. A single polygon was drawn for each lineage group to include all sites assigned to that group (Fig 1). The small disjunct population of bristlecone pine that occurs on the San Francisco Peaks was not evaluated in Schoettle et al. [22], and is included in this analysis as its own group. While Schoettle et al. [22] defined the genetic lineages and the geographic range of lineage groups, we build on this work by defining the suitable climate space for each lineage group. There were 176 plots (out of the 1866 total plots within the geographic range of lineage group 1) with bristlecone pine present in the lineage group 1, 76 plots (out of 747 total plots) in lineage group 2, 119 plots in lineage group 3 (out of 2642 total plots), and 29 plots (38 total plots) in the San Francisco Peaks.

### Suitable climate space

Habitat suitability maps have been used with relatively good success to investigate a variety of scientific issues [37]. These tools statistically relate multiple abiotic habitat characteristics [38] with observed occurrences of a species, thus fitting the original definition of the Hutchinsonian environmental niche [38,39]. While these models are useful for understanding the current conditions associated with species locations, they only reflect a snapshot view of the expected relationship between environmental conditions and species presence. A major assumption is that the modeled species are in pseudo-equilibrium with their environment, an assumption that could be questioned for very long-lived tree species [40]. Although this approach doesn’t explicitly require a mechanistic link between environmental gradients and population fitness [37], this approach can provide key insights into climatic thresholds that drive species success on the landscape.

The Random Forests classification and the regression tree package [41–43] was used to model species presence and absence based on 27 climate surfaces (Table 1) that were developed by Rehfeldt et al. [44]. This nonparametric approach is resistant to over fitting, multicollinearity, and spatial autocorrelation [41]. The regression tree analysis used 10 independent forests of 100 independent regression trees. Random Forests build each tree using a separate bootstrap sample of the data and withholds 36% of the data to compute the classification error. Indices of variable importance (mean decrease in accuracy) were used to drop the 12 least important variables after the first run. The regression procedure was then rerun nine more times with the remaining predictors, dropping the least important 1 to 3 predictors at each run until classification errors began to increase. The Random Forests run with the fewest variables selected prior to detecting an increase in classification error were considered the most parsimonious bioclimatic model for the genetic lineage or species. The estimated climate and projected climates of each location was run down the 100 regression trees in the final set. A single-tree prediction is termed a vote. The votes were averaged and only mean votes greater than or equal to the 50% threshold were considered suitable climate for the species.

**Table 1:** Acronyms and definitions of climatic variables used in Random Forest procedures. Climate variable layers were developed by Rehfeldt et al. [44]. Variables significant in climate models are bolded.

Acronym	Description
**Temperature**	
D100	The sum of degree-days (Julian Date) > 5 °C = 100
DD0	The sum of days > 0°C
DD5	The sum of days > 5°C
FDAY	First freeze day (Julian Date)
FFP	Length of frost free period (days)
MAT	Mean Annual Temperature (°C)
**MMAX**	**Mean Maximum Temperature** (°C)
MTCM	Mean temperature in coldest month (°C)
MTWM	Mean temperature in warmest month (°C)
MMIN	Mean minimum temperature in coldest month (°C)
MINDD0	Negative degree-days using minimum daily temperature for calculation (days)
SDAY	Date (Julian Date) of last freezing temperature in spring
**FDAY**	**Date (Julian Date) of first freezing temperature in autumn**
GSDD5	Mean degree-days > 5°C between SDAY and FDAY. MMAX Mean temperature in warmest month (days)
TDIFF	Summer–winter temperature differential: MTWM-MTCM MAPDD5 (MAP X DD5)/1000 (°C)
**Precipitation**	
MAP	Mean annual precipitation (mm)
GSP	April–September precipitation (mm)
**SMRP**	**Summer Precipitation: July–August precipitation** (mm)
SPRP	Spring Precipitation: April–May precipitation (mm)
WINP	Winter Precipitation: November–February precipitation (mm)
PRATIO	Growing season precipitation balance: GSP/MAP (mm)
SMRPB	Summer Precipitation Balance: (jul + aug+sep)/(apr +may+jun) (mm)
**SMRSPRPB**	**Summer/Spring precipitation balance: (jul+aug)/(apr+may)** (mm)
SDI	Summer dryness index: (GSDD5)0.5/gsp
SDIMINDD0	SDI x MINDD0
**TDGSP**	**TDIFF/GSP** (°C / mm)
**MAPDD5**	**(MAP x MINDD0) / 1000**

Once the suitable climate space was defined under current conditions, climate change projections for the year 2090 were used to estimate changes in suitable climate space. The year 2090 was selected to aid comparison to other western species and forest types that have been evaluated with similar methods [44]. We used a regional summary of the IS92a scenario (1% year^-1^ increase in greenhouse gases after 1990) of the International Panel on Climate Change from General Circulation Models produced by the Canadian Centre for Climate Modeling and Analysis (CGCM2_ghga) [45]. The CGCM2_ghga is generally well respected, and provides an illustration of the potential effects of global warming on species realized climatic niche [45,46]. The regression tree approach has been used to define the realized climate niche for > 70 western tree species [44,46–52] and is used here to model the realized climate niche for bristlecone pine with all lineage groups combined and by individual lineage groups (i.e., lineage groups 1–3 and the San Francisco Peaks).

### Shifts in species and forest types

To understand the potential for bristlecone pine forests to be replaced by other forest types, we merged the suitable climate space for the dominant tree species of important forest types or zones. Douglas fir, subalpine fir, and Englemann spruce constitute the upper subalpine forests. Limber pine and lodgepole pine were used to define the lower subalpine forests. Ponderosa pine and quaking aspen (i.e., lower elevation species) are grouped to represent species encroachment from the montane zone, and pinyon-juniper was evaluated to quantify southwestern lower elevation woodland species encroachment. The current and 2090 probability scores for suitable climate space were obtained from the USDA Forest Service—Moscow Forestry Sciences Laboratory (http://charcoal.cnre.vt.edu/climate/species/). The same random forests classification approach was used to develop these layers [44,47] and probabilities greater than or equal to 50% were considered suitable climate for forest types and zones.

We evaluated changes in the elevational range in climate space for each forest type or zone. Elevation data (ASTER Global DEM) was obtained from the USGS Earth Explorer (https://earthexplorer.usgs.gov). We used changes in the percent overlap with the bristlecone pine climate space to further evaluate the potential for different forest types to encroach on the space occupied by bristlecone pine. The inclusion of additional predictor variables representing the presence–absence of known competitors has been shown to increase the predictive power of models [44,53,54]. By incorporating a measure of the climate space of subalpine forest types, we include information about the physical conditions that are not accounted for by the environmental descriptors included in climate space models. Although this analysis does not conclusively demonstrate competitive exclusion or release, it can provide directional hypotheses that can be tested using observational or experimental field studies.

## Results

### Climatic differences between lineage groups

Average climate conditions varied across and between bristlecone pine lineage groups. Lineage group 1 had the lowest mean annual precipitation and growing season precipitation, while lineage group 2 had the lowest mean annual temperature, mean temperature in the coldest month, July average temperature, and frost-free period (days; Table 2). Lineage group 2 also had the highest growing season precipitation. Lineage group 3 occupied sites that generally had warmer mean annual and July temperatures, longer frost-free periods, and compared to lineage groups 1 and 2, the warmest mean temperature in the coldest month. Climate conditions in the San Francisco Peaks were most similar to lineage group 3 and exhibited the highest mean temperature in the coldest month and mean annual precipitation.

**Table 2:** Mean climatic conditions by lineage group. Mean annual temperature (MAT), mean temperature in the coldest (MTCM) and warmest (MTWM) months, frost free period (FFP), mean annual precipitation (MAP) and growing season precipitation (GSP) were averaged across lineages groups. Standard deviation within lineage groups are listed in parenthesis.

Lineage Group	MAT (°C)	MTCM (°C)	MTWM (°C)	FFP (days)	MAP (mm)	GSP (mm)
1	2.95 (2.5)	-8.49 (1.9)	14.55 (2.9)	56.49 (29.8)	445.08 (226.6)	261.81(86)
2	-0.25 (1.9)	-11.01 (2.1)	11.82 (1.6)	37.92 (10.6)	584.16 (124.8)	321.55 (62.8)
3	6.23 (2.9)	-4.22 (2.5)	17.40 (3.5)	91.89 (34.5)	466.43 (133.8)	314.54 (72.9)
San Francisco Peaks	5.00 (1.9)	-3.97 (1.2)	15.78 (2.2)	72.82 (14.5)	604.95 (117.5)	285.03 (42)

### Suitable climate space

**Fig 2:**
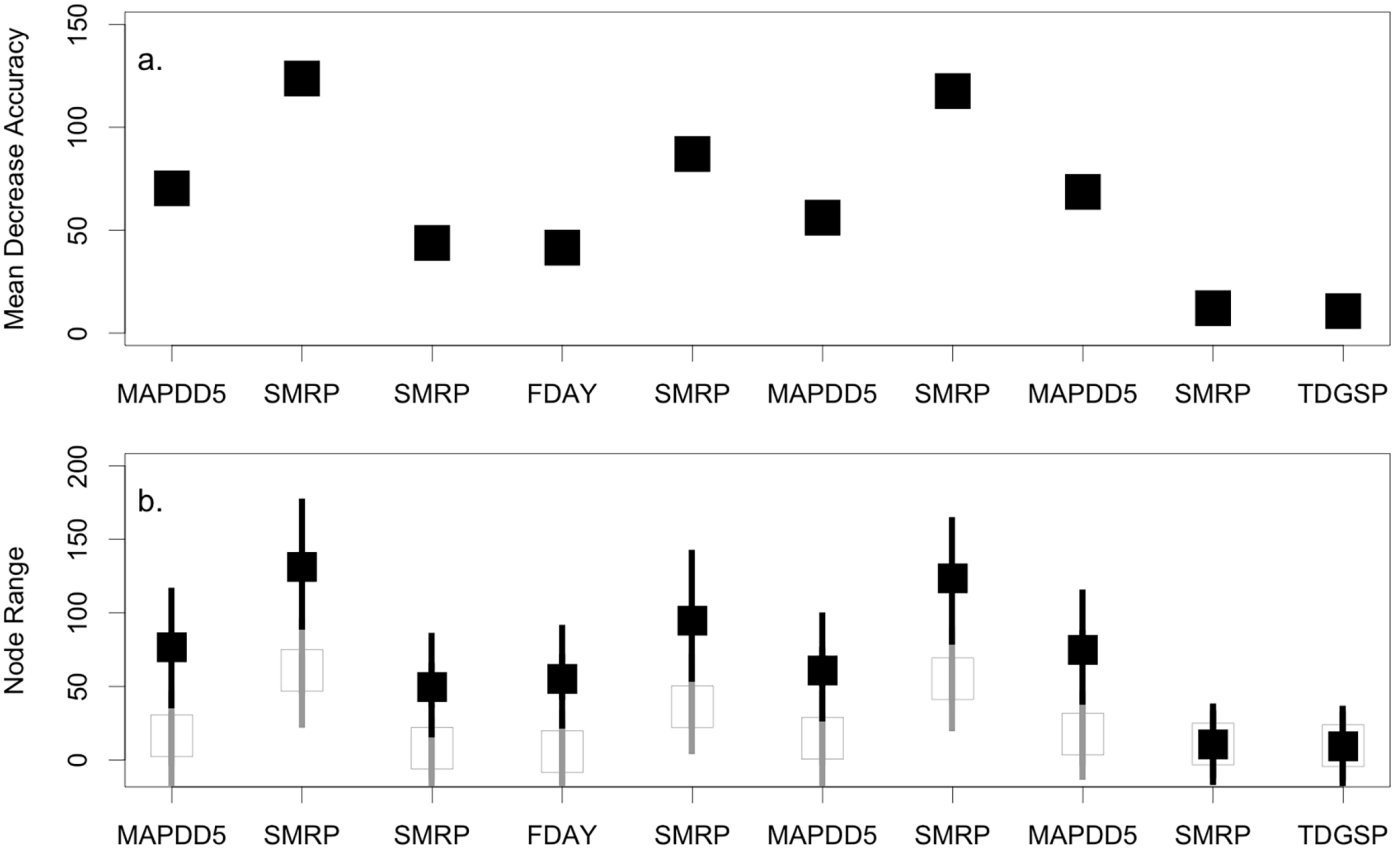
Variable importance plots for the forests used to define the bristlecone pine climate space. Variables with a large (a) mean decrease in accuracy are more important for classification. (b.) The node range for variables show the range in node values that denote presence (black) and absence (grey) of bristlecone pine for each variable.

**Fig 3:**
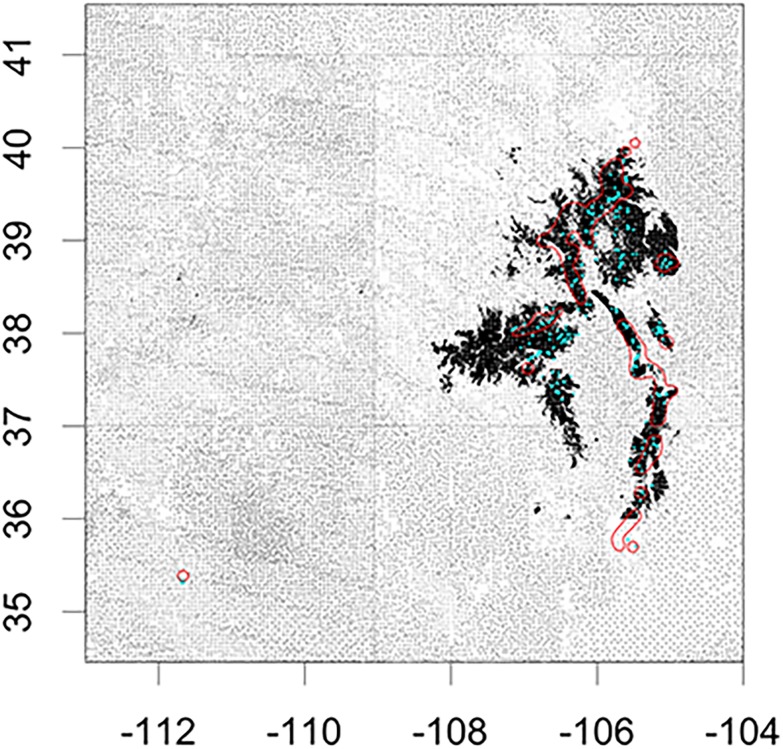
The bristlecone pine range (Little 1971; red) and the current suitable climate space (black) overlapped 20%. Plot level data (grey) showed that areas where bristlecone pine was present (blue; n = 399) was in agreement (98% accuracy) with the suitable climate space.

Both temperature and precipitation were important for defining the suitable climate space for bristlecone pine (Fig 2). The random forests classification tree procedure produced 2-variable algorithms that accurately predicted the presence of bristlecone pine (Accuracy = 99%; Fig 2). Most of the errors were due to erroneous predictions of presence (i.e., errors of commission = 98% of errors). Models for all lineage groups together and lineage groups 2 and 3 included the interaction between mean annual precipitation and the sum of days > 5°C (MAPDD5) and the summer precipitation balance (SMRP). For lineage group 1, the summer precipitation balance and the first freeze day (FDAY) defined the suitable climate space. The model for the San Francisco Peaks differed from the other lineage groups and did not contain a temperature parameter. The summer precipitation balance and the summer-winter temperature differential / growing season precipitation (TDGSP) defined the suitable climate space for the San Francisco Peaks. A common thread across models was a high mean decrease in accuracy for SMRP (Fig 3). Variable importance was second highest for MAPDD5, followed by FDAY, and was the lowest for TDGSP. Variable importance was low and differences in node range was small for the San Francisco Peaks model, which may reflect the low sample size available for the analysis (Fig 2).

**Fig 4:**
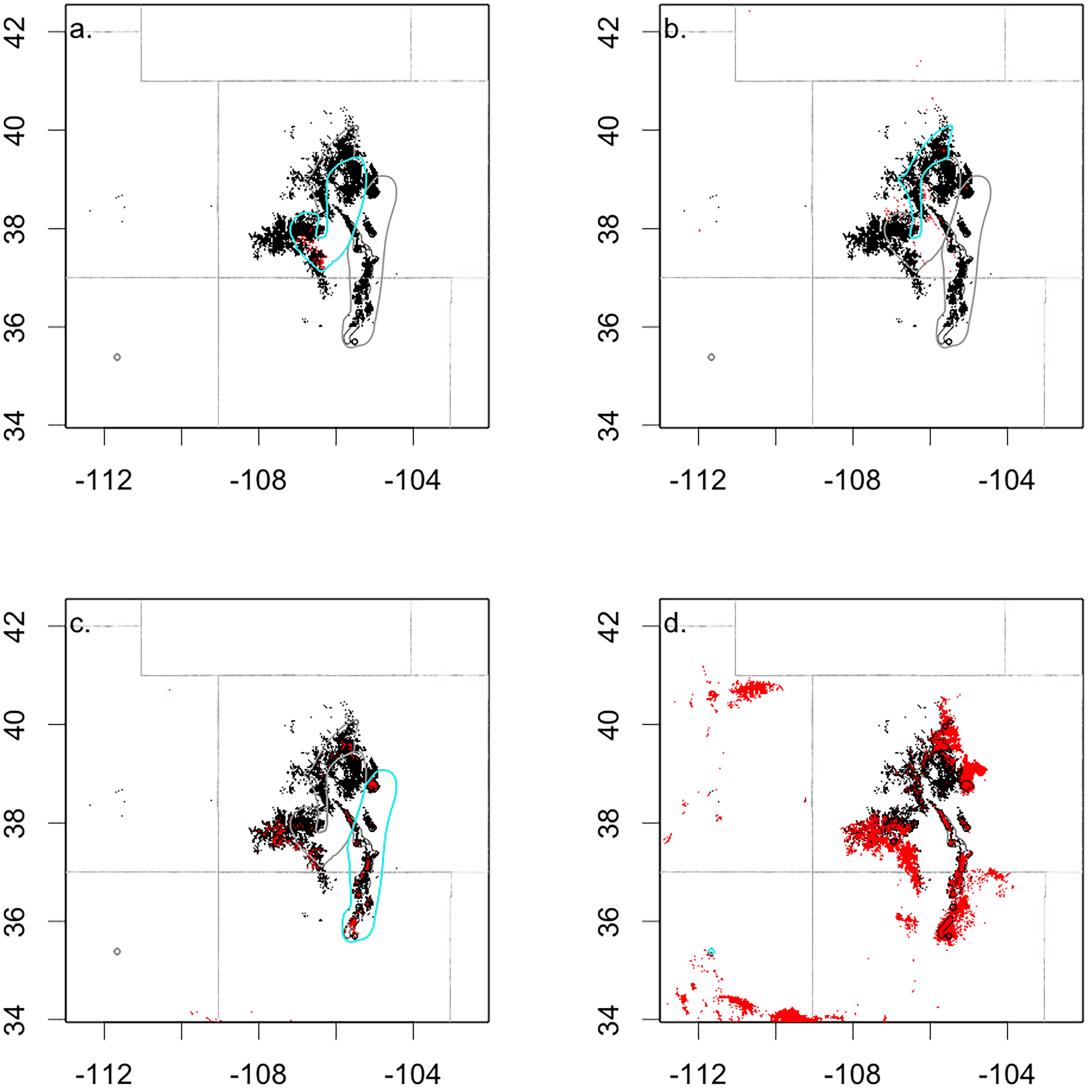
The current climate space (black) versus the 2090 climate space (red) for (a.) lineage group 1, (b.) lineage group 2, (c.) lineage group 3, and (d.) the San Francisco Peaks. The current distribution of each lineage group is outlined in blue in each respective map.

The bristlecone pine suitable climate space was concentrated in Colorado and New Mexico (71%; Fig 3). Although the range defined by Little [31] for bristlecone pine identified general areas where the species was found, this coverage overlapped just 20% with the current climate space. Of the 399 plots in Colorado, New Mexico, and Arizona with bristlecone pine present, 98% of those plots were within the suitable climate space. Under current conditions, there was a 30% overlap in the suitable climate space of all lineage groups (Fig 4). The majority of the climate space for all lineage groups combined intersected the area dominated by lineage group 3 (90%), 70% intersected lineage group 2, and 30% intersected lineage group 1. A large portion of the current climate space for all lineage groups combined also intersected data from the San Francisco Peaks (70%). Across all lineage groups, the majority of plots that contained bristlecone pine were on National Forest land (84%) along with most of the current suitable climate space (74%).

### Climate change projections

Changes in mean annual temperature (MAT; 4 ± 0.001 °C) and precipitation (MAP; 29 ± 0.08 mm) co-occur with shifts in maximum temperature (MMAX; 3.3 ± 0.001 °C), MAPDD5 (481 ± 0.51 mm), and the summer precipitation balance (SMRP; 8.4 ± 0.03 mm) in the 2090 climate layers. There is also a 21 day (± 0.02) delay in the Julian date of the first freezing temperature in autumn and on average, growing season precipitation increases 7.4 mm (± 0.05) in 2090 compared to current conditions.

**Fig 5:**
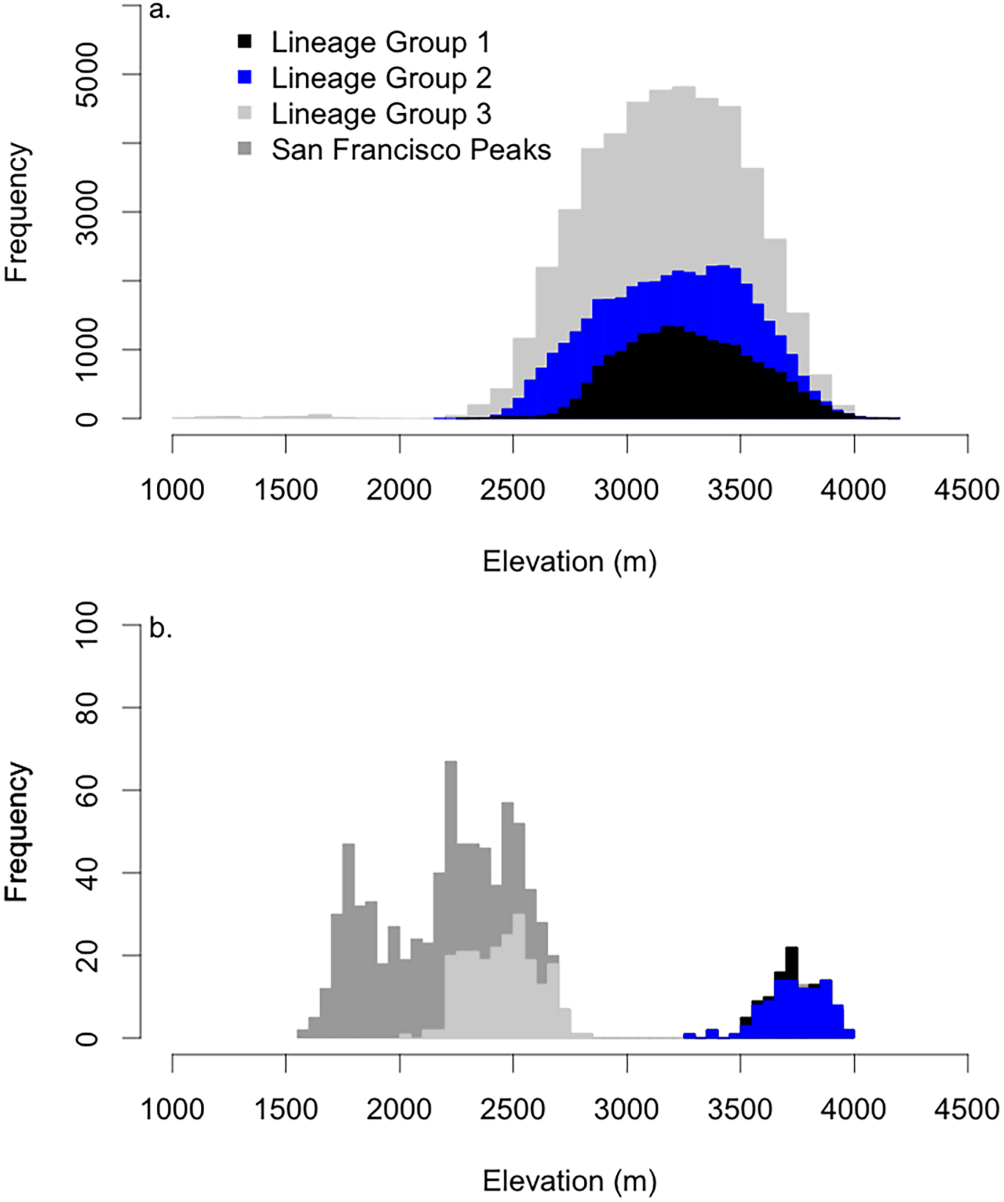
The frequency of (a) contraction and (b) expansion in bristlecone pine climate space by lineage group and elevation in 2090.

**Fig 6:**
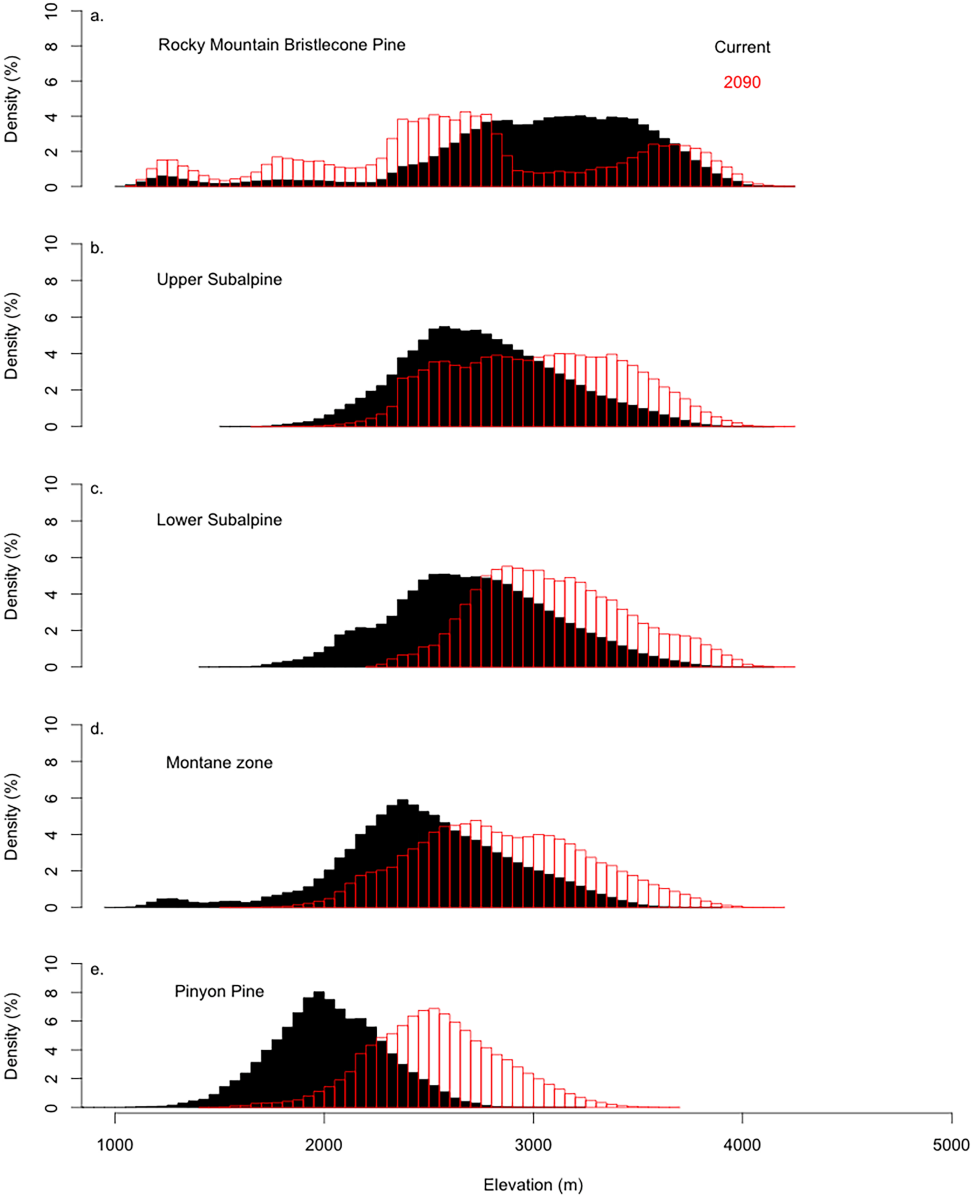
The density of the current (black) and future (red) climate space by elevation for bristlecone pine (model for all lineage groups), upper subalpine, lower subalpine, montane zone, and pinyon pine forests in the Southern Rockies.

Our models project a 74% decrease in the bristlecone pine suitable climate space in 2090 (Figs 3 and 4). Although the climate space declines substantially, overlap between the three lineage groups is maintained at 26%. Comparing the 2090 model for all lineage groups with models for individual lineage groups we estimate that, the future climate space for the San Francisco Peaks expands in 2090 and occupies the greatest area (99% of the 2090 climate space defined by all lineage group model), followed by lineage group 3 (35%), lineage group 2 (2%), and lineage group 1 (<1%). The 2090 climate space for lineage group 1 is compressed in the southern portion of the current distribution (Fig 4a). While we project substantial declines in the 2090 climate space for all three lineage groups in the species’ core distribution, the climate space associated with the San Francisco Peaks expands (Fig 5b). The majority of the bristlecone pine 2090 climate space overlaps with the current bristlecone pine forest (90%), while just 10% occurs in new areas (Figs 4 and 5a). Small gains at high elevations (>3000 m) are projected for lineage groups 1 and 2 (Fig 6b). Between an elevation of 2000–2750 m there is expansion in the geographic distribution of the climate space for lineage group 3, and the climate space associated with the San Francisco Peaks population expands at 1000–2700 m (Fig 6b). The future projection of the San Francisco Peak source may however be an over estimate as it was not constrained by temperature; the lack of a temperature parameter in the San Francisco Peaks model may have been an outcome of the narrow distribution of this disjunct population. Similar to patterns observed in the current climate space, the majority of the 2090 climate space (94%) continues to occur on National Forest land.

### Shifts in species and forest types

Under current conditions, the climate space for upper subalpine forests has the greatest overlap with the bristlecone pine climate space (81%), followed by the climate space for lower subalpine forests (68%), species from the montane zone (67%) and pinyon pine woodlands (6%). All groups’ climate space overlapped with 45% of the current bristlecone pine climate space. The climate space for the lower elevation forest types was most frequent between 2500 and 3000 m under current conditions (Fig 6). In 2090, the climate space for all forest types declines by (13%) throughout the southern Rockies and distributions shift towards higher elevations (Fig 6). The greatest shifts in elevational distributions of forest type climate space occurs in the montane zone (i.e., ponderosa pine and aspen) and in lower subalpine species (i.e., limber pine and lodgepole pine) (Fig 6). By 2090 the bristlecone pine climate space is entirely shared with the climate space of other forest types. The distribution for lower elevation species’ climate space shifts up in elevation, while bristlecone pine climate space is not present and overlap declines for all forest types climate space except for pinyon pine (+21%). The 2090 climate space for lower elevation species is more frequent between 3000m and 3500m, which coincides with declines in 2090 bristlecone pine climate space.

## Discussion

This research used bristlecone pine population genetics [22], geographic distribution, and suitable climate space to evaluate the species’ potential vulnerability to climate change in 2090. We hypothesized that (1) the future climate space for bristlecone pine would decline; (2) the climate space for lineage group 3 would be more extensive than the climate space for groups 1 or 2; and (3) the climate space for lower elevation species would encroach on the area that is currently occupied by bristlecone pine. Results support all three hypotheses, indicating that significant reductions in the bristlecone pine climate space and the expansion of the climate space for lower elevation species into higher elevations may reduce the future bristlecone pine range.

### Abiotic versus biotic pressure

One general pattern of range limitation is the relative importance of abiotic versus biotic limiting factors in ecological gradients [55]. Currently, abiotic limitations (i.e., maximum temperatures, seasonal precipitation patterns, and disturbance regimes) constrain upward expansion of bristlecone pine forests [56], while biotic limitations (i.e., competition from lower elevation species) have a greater influence on persistence at mid-elevations and limit downward expansion. Like most other species [55], one direction is physically stressful (higher elevation) and the other is biologically stressful (lower elevation) for bristlecone pine. Although climate change is likely to reduce upward physical stress (e.g., minimum temperature), this is expected to be offset by a corresponding increase in competitive stress from lower elevation species that will represent a larger portion of the species composition in the subalpine zone (Fig 6) [57].

Bristlecone pine appears to be excluded from climate space specific to other species (Fig 5). While competitive interactions are not explicitly modeled here, overlapping climate space for co-occurring and neighboring tree species might reflect competitive pressures (Fig 6) [58,59]. Suitable 2090 climate space for bristlecone pine exhibits a bimodal distribution along the elevation gradient in the future presumably due to persistence at lower elevations in the Southern Rocky Mountains and exclusion at the mid-elevations by changes in climatic conditions that favor other lower elevation species. This pattern can be seen currently along the eastern margins of South Park and the Sawatch range in central Colorado, where bristlecone pine coexists with ponderosa pine at low elevations, is absent from portions of the upper montane and subalpine zones, then reoccurs near alpine treeline [60]. Historical patterns of species distributions along the elevational gradient further suggests the role of competition in the distribution of five-needle pines [61]. Pollen and charcoal data from the Yellowstone region suggest that five-needle pine species, now restricted to higher elevations, were abundant across broader elevational ranges during a period when Engelmann spruce and lodgepole pine were less common [62]. These shifts have strong implications for changes in fire regimes, vulnerability to pest and pathogens, and altered C dynamics across the Southern Rockies.

### Climate change and disturbance regimes

Climate change is expected to increase the number of fires at high elevations in the southern Rockies [63,64], resulting in a decline in the abundance of older forests [29]. As an early seral species, bristlecone pine forest structure is currently thought to be the result of long fire return intervals, time since fire, and dispersal limitations and modified by smaller intra-stand disturbances [29,30]. Shifts in the location of suitable climate, the increase in lower elevation species presence that have different fire regimes, and recovery patterns in bristlecone pine forest might alter the role of fire in promoting bristlecone pine forests in the future. With these shifts in forest composition, fire return intervals are likely to be greatly reduced (i.e. increasing fire frequency), which might promote novel forest development throughout the current bristlecone pine range.

An increase in fire frequency may promote regeneration of bristlecone pine, increasing the adaptive capacity of the species [65]. Diversifying age-class structure, particularly through increased abundance of younger cohorts, may serve the dual purposes of 1) facilitating more rapid selection for adaptive traits [17,66,67], and 2) ensuring the presence of many small-diameter individuals less vulnerable to drought stress, and more likely to survive mountain pine beetle (*Dendroctonus ponderosae* Hopkins) attack [60]. However, an increase in fire frequency and a smaller climate space suitable for bristlecone pine at the high elevations may reduce the likelihood of bristlecone pine trees achieving exceptional longevity (>1500 years) in the future.

Bristlecone pine is confronted by another directional selection threat—the lethal disease white pine blister rust (WPBR). The southern Rocky Mountains are at the infection front of the non-native pathogen *Cronartium ribicola* J. C. Fisch. that causes WPBR [68]. Bristlecone pine is susceptible to WPBR [69,70] and the disease is expected to continue to spread through much of the bristlecone pine distribution [71]. Currently, the disease has only been found on bristlecone pine in south-central Colorado [72] in the area that contains lineage group 3 [22]. Genetic resistance to WPBR is not distributed uniformly within the five-needle pine species, including bristlecone pine [22], and how disease resistance traits are associated with abiotic stress tolerances is unclear but may be significant [73,74]. Consequently, the climate space of five-needle pines may be shifting under directional selection for resistance to WPBR [73,74], further complicating climate change projections. Range-wide genetic collections (seed and tissues) of bristlecone pine have been made and these efforts are the first proactive coordinated range-wide genetic collection design and forest health assessment for a threatened, but not yet heavily impacted, tree species [21]. The 2090 climate space projections presented here provide further insights to guide proactive management to increase the resilience of the threatened populations [66] and consider assisted migration of WPBR resistant genotypes to areas with suitable 2090 climate space.

Recent threats from the expanding distribution of native bark beetle infestations [75] may also affect future tree species range distributions. This may be of particular concern if a greater proportion of bristlecone pines distribution is restricted to lower elevations in the future as projected by the results of this study (Fig 6). However, the other species that may be excluding bristlecone from the higher elevation climate spaces in the future also have uncertain futures in the presence of expanding and intensifying bark beetle epidemics.

### Limitations to understanding climate change impacts on species range shifts

The climate spaces of specific genetic lineage groups for bristlecone pine shifted differently under a climate change scenario, with proportional representation of each climate space changing in the future. The implication of this shift on genetic diversity patterns is complex. Populations can respond to environmental change by moving to a new area corresponding to environmental conditions for which they are adapted [15], or through phenotypic plasticity and/or genetic adaptation to the new conditions, or combinations of these responses [17]. The inferences made in the current study to future species distribution and genetic structure assume only unimpeded population migration and no other evolutionary processes. Persistence, via plasticity or adaptation, on sites with climates that are no longer suitable is not considered in the model, suggesting that the projected range contraction for the 2090 bristlecone pine climate space may be unrealistically severe. Due to the stress tolerance and long life span of bristlecone pine, individual trees may persist in suboptimal environments for centuries, yet without regeneration populations are not adaptable or sustainable into the future. Plant species distributions that are in climatic disequilibrium might lead to an increased potential for extinction within all or portions of species’ ranges. This, combined with long fire return intervals, could be the cause of the lack of regeneration in some bristlecone pine forests [29]. The low genetic diversity, delayed reproductive maturity, protracted regeneration dynamic, and slow growth of bristlecone pine suggests a low adaptive potential to rapidly changing conditions [22].

## Conclusions

Although the availability of bristlecone pine climate space is projected to decline substantially in 2090, availability of suitable climate space for lineage group 3 and for populations from the San Francisco Peaks suggest that these populations are likely to be the most suitable for the future climate. While these populations may be of particular management interest, current populations of lineage groups whose 2090 climate space contracts (lineage group 1 and 2) may be of particular conservation interest. Considering that the majority of the current and future suitable climate space exists on Forest Service land, the future of bristlecone pine forests is essentially within the jurisdiction of the United States Forest Service. With management intervention, assisted migration is possible and this analysis provides insights into suitable genetic lineage groups (i.e. lineage group 3 and populations from the San Francisco Peaks) that may be considered for source material. Although this approach provides an important and novel step beyond models that assume a species is genetically homogenous, further integration of known evolutionary processes are needed in climate change response modeling [14].

## Supporting information

S1 Fig (a.) Differences in elevation between true and public Forest Inventory and Analysis (FIA) plot locations. (b.) A comparison of the bristlecone pine climate space defined by true (yellow) and public FIA plots combined with supplemental plots.
(TIF)
