# Supplementary figures and images for: The future of subalpine forests in the Southern Rocky Mountains: Trajectories for Pinus aristata genetic lineages
Source: PLoS One. 2018 Mar 19;13(3):e0193481. doi: 10.1371/journal.pone.0193481 (PMC5858753; doi:10.1371/journal.pone.0193481)

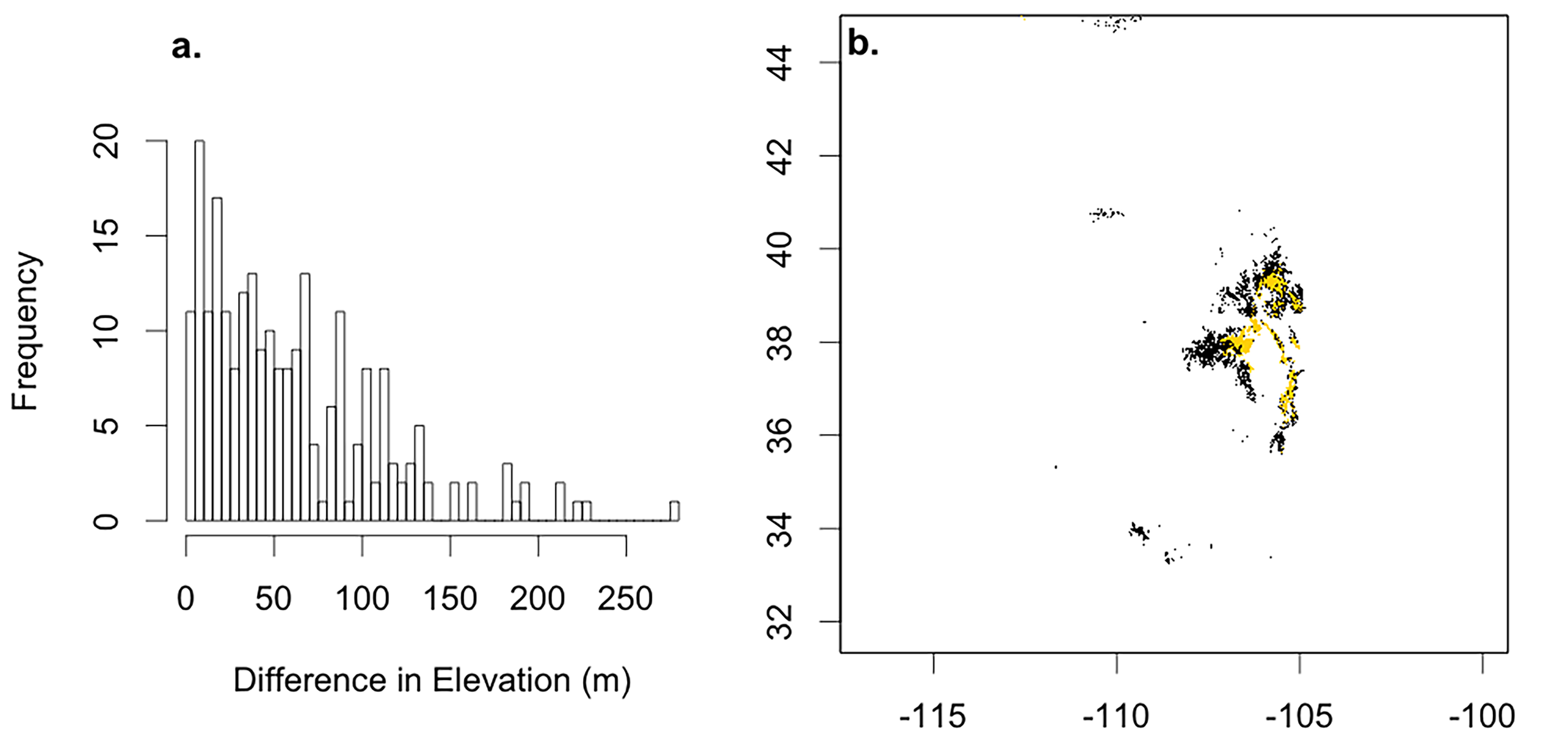

Supplement: S1 Fig — (a.) Differences in elevation between true and public Forest Inventory and Analysis (FIA) plot locations. (b.) A comparison of the bristlecone pine climate space defined by true (yellow) and public FIA plots combined with supplemental plots. (TIF) [file pone.0193481.s001.tif]
